# High-Throughput Method for Automated Colony and Cell Counting by Digital Image Analysis Based on Edge Detection

**DOI:** 10.1371/journal.pone.0148469

**Published:** 2016-02-05

**Authors:** Priya Choudhry

**Affiliations:** Department of Chemistry, California Institute of Technology, Pasadena, California, United States of America; Queensland University of Technology, AUSTRALIA

## Abstract

Counting cells and colonies is an integral part of high-throughput screens and quantitative cellular assays. Due to its subjective and time-intensive nature, manual counting has hindered the adoption of cellular assays such as tumor spheroid formation in high-throughput screens. The objective of this study was to develop an automated method for quick and reliable counting of cells and colonies from digital images. For this purpose, I developed an ImageJ macro Cell Colony Edge and a CellProfiler Pipeline Cell Colony Counting, and compared them to other open-source digital methods and manual counts. The ImageJ macro Cell Colony Edge is valuable in counting cells and colonies, and measuring their area, volume, morphology, and intensity. In this study, I demonstrate that Cell Colony Edge is superior to other open-source methods, in speed, accuracy and applicability to diverse cellular assays. It can fulfill the need to automate colony/cell counting in high-throughput screens, colony forming assays, and cellular assays.

## Introduction

The analyses of shape, number, color, size and morphology of cells and tissues has made significant contributions to our understanding of botany, zoology, genetics, and evolution[1–9]. Properties such as cell shape, cell movement, tissue shape, protein expression, percentage of stained cells, and colony formation are commonly measured and analyzed in microbiology, immunology, cellular and molecular biology[4, 5, 9–14]. Traditionally these measurements have been done manually, making them time-consuming and subjective. In recent years, improved accessibility of digital cameras has given us the opportunity to automate the image analysis step, making it faster and less subjective. Several softwares and macros are available for such analysis. The aim of this report is to introduce a new ImageJ macro and compare it to existing open-source tools, for common applications such as spheroid measurements, clonogenic assays, and counting bacterial cells and colonies.

Counting cell colonies is essential for estimating microbial content [15, 16], measuring cytotoxicity [17, 18] and the function of specific genes in microbiology, immunology, and cell biology [19–22]. For example, in cancer biology, the effect of radiation is measured using the clonogenic colony formation assay [23–28] and the proportion of brain tumor initiating cells is quantified using the neurosphere formation assay [29–31] (tumorsphere assay for other cancer cells [32–35]). While manual counting remains the gold standard, this process has low reproducibility, is slow, tedious and inadequate for high throughput assays.

While automation provides speed, accuracy and reproducibility, it is not straightforward and errors can be introduced at various steps. Currently available macros delineate colonies in two steps: 1) *thresholding* divides the image into foreground and background, and 2) *segmentation* separates colonies that are overlapping or in contact with each other. In addition, artifacts such as cell debris, bubbles, edges of culture dishes, and agar/media clumps must be excluded during the analysis. Such exclusion is commonly performed using *denoising* algorithms. Parameters for denoising have to be chosen carefully based on the images. Colony detection can be affected by numerous parameters related to the image (size, resolution, sample lighting and contrast), and the colony (size, shape, clustering, overlap and location near the periphery). Furthermore, imaging programs must be flexible with tunable parameters defined by the user (eg., colony size).

There are several commercially available tools for measuring colonies (ColonyDoc-It^™^ and AIDBacSpot for Bacterial colonies) and tumor spheroidss (Nexelcom’s Celigo and VisionGauge from Visionx). However, these programs are proprietary and sometimes require the purchase of matching equipment. This makes the tools expensive and hence restrictive. I will compare several open-source methods to count colonies from digital images. The NICE (NIST’s Integrated Colony Enumerator) software uses a combination of thresholding and extended minima to count colonies[36]. The circular Hough image transform algorithm (CHiTA) pre-processes images by a combination of erosion and Gaussian smoothing, and then identifies colony edges by intensity gradient field discrimination[37]. However, both NICE and CHiTA run on MATLAB, making them less accessible and user-friendly. Since many users may not have a deep understanding of image processing, the parameters might not be intuitive. Here, I will compare and determine the merits of developed macros/pipelines on publically available, commonly used and user-friendly programs such as ImageJ, Cell Profiler and OpenCFU.

OpenCFU is a software with GUI, created to be faster, more intuitive, and more accurate than NICE[38]. It is open-source and the user can define a folder of images that need to be processed, in a single step. ImageJ is a fast, publically available, Java-based image processing program developed at the National Institutes of Health with its own GUI. It has a large and knowledgeable user community, and extensive plugins and macros for specific purposes. Specifically, Cai et al. published an ImageJ macro for colony detection and measurements, which uses a combination of thresholding, watershed segmentation and particle measurements [28]. However, unstained colonies such as bacterial agar colonies often do not differ greatly in color/contrast from background. Hence, thresholding does not provide appropriate distinction between colonies and background.

**Fig 1:**
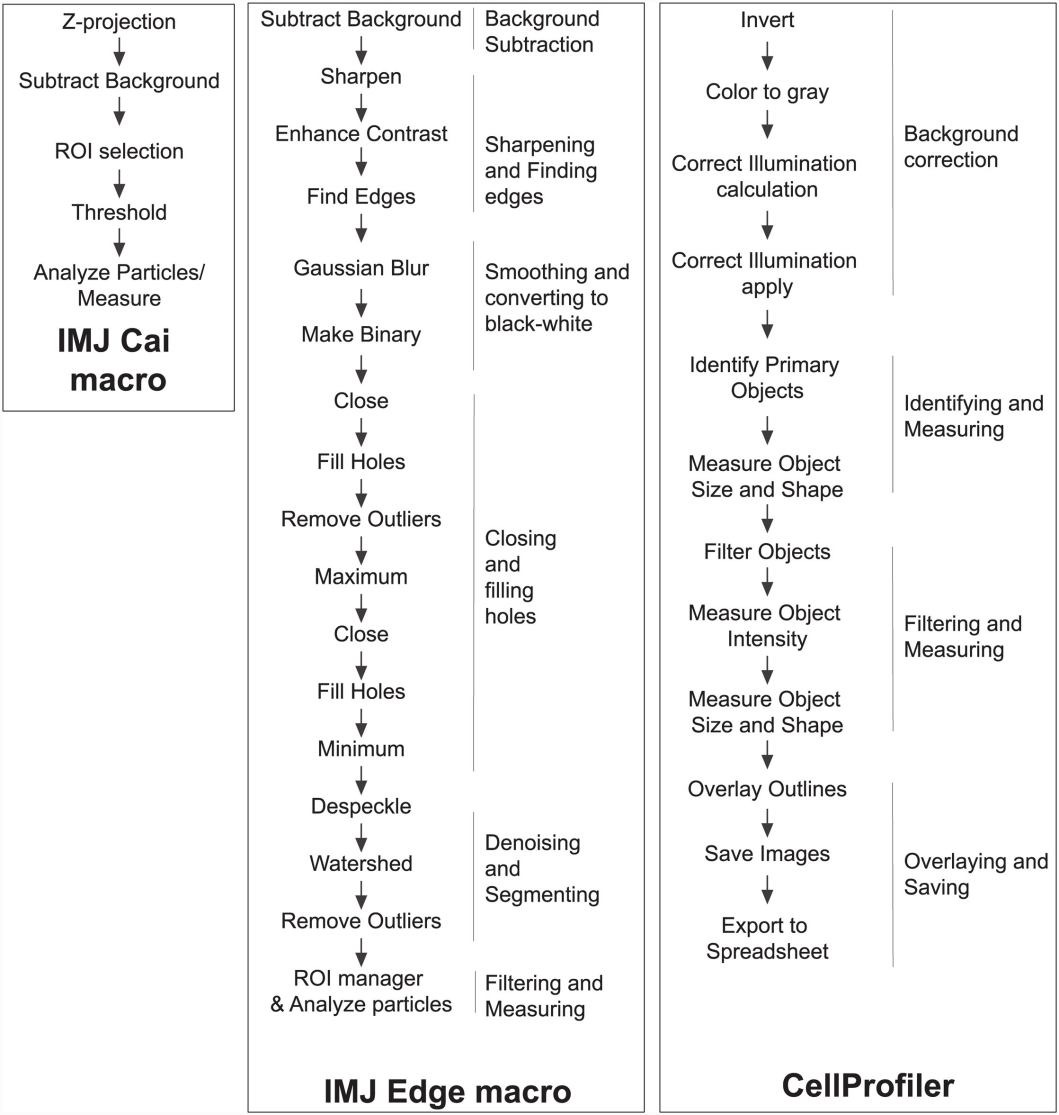
Flowchart representing the processing steps in different Colony detection methods. OpenCFU’s flowchart is not shown here.

CellProfiler is another commonly used program that was designed for biologists with minimal programming knowledge to measure biological phenotypes quantitatively[39]. Similar to ImageJ, algorithms for image analysis are available as individual modules that can be placed in sequential order to create a pipeline. Several commonly used pipelines are available for download, and can be used to detect and measure biological objects. I developed a new pipeline Cell Colony Counting, for measurement of unstained cells and colonies (Fig 1) and will make it available with the publication of this article. Since this is the only CellProfiler Pipeline tested in this work, I will refer to it as CellProfiler for simplicity.

In this work, I present an alternative ImageJ macro IMJ Cell Colony Edge that relies on edge detection, Gaussian smoothing and filling of holes for colony detection (Fig 1, S1 Appendix). For simplicity it will be referred to as IMJ Edge. I further demonstrate that this method is fast and accurate, and works on a variety of images (bacterial, clonogenic, spheroid). It is tunable, both in steps and in parameters like colony size. Like other ImageJ macros, it can be easily run in batch mode to analyze a folder of images quickly, hence enabling high-throughput colony/cell counting and measurements.

## Materials and Methods

### Cell Culture

All experiments were performed under standard cell culture conditions at 37°C and 5% CO_2_. The human breast cancer line T47D was purchased from the American Type Culture Collection (Manassas, VA). Cells were cultured in RPMI 1640 medium (Sigma-Aldrich, St Louis) supplemented with 10% fetal bovine serum (Invitrogen, Carlsbad) and 1% penicillin-streptomycin (Gibco-Invitrogen Inc). For tumorsphere assays, 25,000 cells were grown in 3% methylcellulose (Sigma-Aldrich) in triplicate in 6-well dishes coated with Poly (2-hydroxyethyl methacrylate) (Sigma-Aldrich). Imaging was done after two weeks of culture. For clonogenic assays, 10^5^ T47D cells were grown overnight and exposed to 0–8 Gy irradiation. A total of 1,000 cells were seeded in triplicate into 6-well tissue culture plates.

### Bacterial cell plating

Escherichia coli were grown on 100mm LB plates at various dilutions overnight at 37°C.

### IMJ Cell Colony Edge

The general IMJ Edge macro with prompts for user input is given in S1 Appendix. It is also available for download from https://sourceforge.net/projects/cell-colony-edge/files/. The parameters used for different analyses are also given. It is quite long, but the user can customize and simplify it for their purposes. Customized versions of IMJ Edge for different images and resolutions (for example, bacterial *versus* clonogenic assay), and are given in S2 Appendix. The tunable values such as minimum and maximum size were based on manual measurements, and are shown in red. S1 Fig shows an example of how tunable parameters are determined by trial and error for an image of a single cell (courtesy [40]). S3 Appendix gives the steps for manual trial and error using the ImageJ GUI. S4 Appendix gives instructions for downloading and using the macro.

The macro has six major steps: 1) Background subtraction, 2) Sharpening and Finding Edges, 3) Smoothing and converting to black and white, 4) Closing and filling holes, 5) Denoising and segmenting, 6) Filtering and Measuring (Fig 1). 1) First, the background is subtracted to enhance contrast and reduce effects of uneven illumination (Figure B in S1 Fig). The radius for Background subtraction was determined empirically. A starting number can be the average radius of colonies. 2) Since the macro relies on edge detection, the next step is sharpening and enhancing the image, followed by finding the edges (Figure C in S1 Fig). ImageJ’s in-built Sobel filter is used in the macro. Users are welcome to download and use Canny edge detection or LoG filter. 3) The image is smoothened (Gaussian Blur), and converted to black and white (Make Binary) (Figure D in S1 Fig). Alternatively, the image can be smoothened by sequential dilate and erode steps. 4) This is followed by closing of the edges to form closed circle/ellipses (Close), closed objects are filled black (Fill Holes), resulting in images containing black colonies on a white background (Figure D in S1 Fig). To ensure that all colonies are detected, an additional step of closing and filling holes is performed. Here, the size of each pixel is increased (Maximum) (Figure E in S1 Fig), in order to bring the detected edges closer to each other, followed by closing and filling (Figure F in S1 Fig). This step allows the detection of colonies whose entire edge along the perimeter fails to be otherwise detected. After filling, the size of the pixels is reduced (Minimum), returning the colony size to their original values (Figure G in S1 Fig). The pixel sizes for this step have to be chosen manually based on the resolution of the images and the size of the colonies. Note that these expanding and shrinking steps were not required for measurement of bacterial colonies.

5) The final steps involve denoising and segmentation, to remove small particles (artifacts) and to separate clustered colonies. S1 Fig focuses on a single cell for clarity, and hence denoising and segmentation steps are not shown. Denoising is done by deselection of particles (Remove Outliers) smaller than a manually set threshold. This step can be performed multiple times with different thresholds. For example, due to small size of bacterial colonies, artefacts such as scratch marks on the plate become very prominent during the closing and filling holes steps. Hence a couple of Remove Outliers steps were inserted before the smoothing step to exclude these false positives (S2 Appendix). 6) Finally, the objects are filtered based on size, circularity, closeness to edge etc, and only objects meeting the user defined criteria are measured. For intensity measurements, such as in clonogenic assays, the ROI’s (Regions Of Interest) can be redirected to the original image, via the ROI manager, and then measured (S1 Appendix).

### CellProfiler Pipeline: Cell Colony Counting

A new pipeline was made for detection of cells and colonies from brightfield/nomarski images (https://sourceforge.net/projects/cell-colony-edge/files/). This pipeline has four major steps: 1) Background correction, 2) Colony detection & Filtering, 3) Measuring Colony parameters, 4) Overlaying and saving images (Fig 1). Background correction is done through its own inherent modules- Color to Gray, Correct Illumination calculation, and Correct Illumination Apply. Modules for object detection (Identify Primary Objects) based on thresholding are available in Cell Profiler. While ImageJ allows filtering at the measuring stage, the primary objects have to be filtered first in Cell Profiler. Hence, the subsequent steps involved measuring the size and shape of the object, followed by filtration based on form factor and size. After filtering, the object’s size, shape and intensity are measured again, outlines of filtered objects are overlaid on the original image and saved.

### Image Acquisition and Analysis

Images of tumorspheres were acquired using brightfield microscopy with a 5X objective (Nikon C1si, Nikon Instruments Inc., Melville, NY, USA). Bacterial plates and 6-well plates in clonogenic assays were imaged using a digital camera that could capture the entire plate area in a single image. Images were were cropped and saved as a folder of.tiff files. For clonogenic assays, the area outside the plate was set to background color. The scale of images was determined using a calibration slide. Images were analyzed using the open-source softwares ImageJ (Fiji package)–IMJ Cai and IMJ Edge macro, OpenCFU and CellProfiler. For manual measurements of colony area and perimeter, ImageJ’s ‘draw ellipse’ and ‘measure’ (Analyze-Measure) tools were used for each colony on the original image. The optimal parameters were determined for each method by trial and error, and measurements of detected objects recorded. The parameters for IMJ Cai were optimized manually as per directions provided in Cai et al [28]. For each method (including manual), images were analyzed five times, and the average values used. To compare the accuracy of various methods, the measurements of the detected objects from various methods were compared to manual measurements.

Detected objects from various methods were matched manually by either printing the labeled processed images, using the x-y coordinates and area measurements, or selecting colonies in the software. If an automated method segmented a colony into parts, then the largest segment was matched with the manual measurement of the full colony. The smaller colony was given an additional colony number. If an automated method did not segment two touching colonies, then that merged colony was matched to the larger of the two manually segmented colonies. The smaller of the two manually segmented colonies was counted as an additional colony, undetected by that automated method. Results from area and perimeter measurement were analyzed in MS Excel. Boxplots were made in R.

## Results

### Tumor spheroid measurements

The use of multicellular tumor spheroids (MCTS) has become very popular in cancer biology. MCTS are formed by inducing aggregation of tumor cells, using various methods such as low-adhesion U-bottom plates and centrifugation, hanging drop or matrix embedding. MCTS have been reported to be the closest available *in vitro* model of small avascular tumors[41]. They have proved especially useful in testing the effect of drugs as they mimic the complex intercellular and cell-extracellular matrix interactions, as well as nutrient and oxygen gradients found in tumors[11, 42–45]. There is increasing evidence that MCTS studies are more predictive of *in vivo* study outcomes than 2D cell culture formats. Despite advances in MCTS culture methods that have facilitated their application in high-throughput drug screening, analysis of the data generated in these assays, specifically images, still constitutes a major bottleneck.

In addition to MCTS, tumors are also assessed by the number of tumor-initiating cells (TICs) they carry. TICs have the ability to self-renew, initiate cancer, and give rise to more differentiated tumor cells when transplanted into immune-compromised mice[46–48]. Tumor cells are grown in serum-free, non-adherent conditions in order to enrich the TIC/progenitor cell population. Only TICs can survive and proliferate under these conditions to form tumorspheres. This assay has been developed from the neurosphere assay for culturing neural stem cells *in vitro*. Hence, the number and size of tumorspheres are key parameters that need to be accurately measured in this assay. Therefore, in this report, I utilized images of tumorspheres to compare various image analysis programs. However, the same methods can also be used for analysis of MCTS and neurospheres.

**Fig 2:**
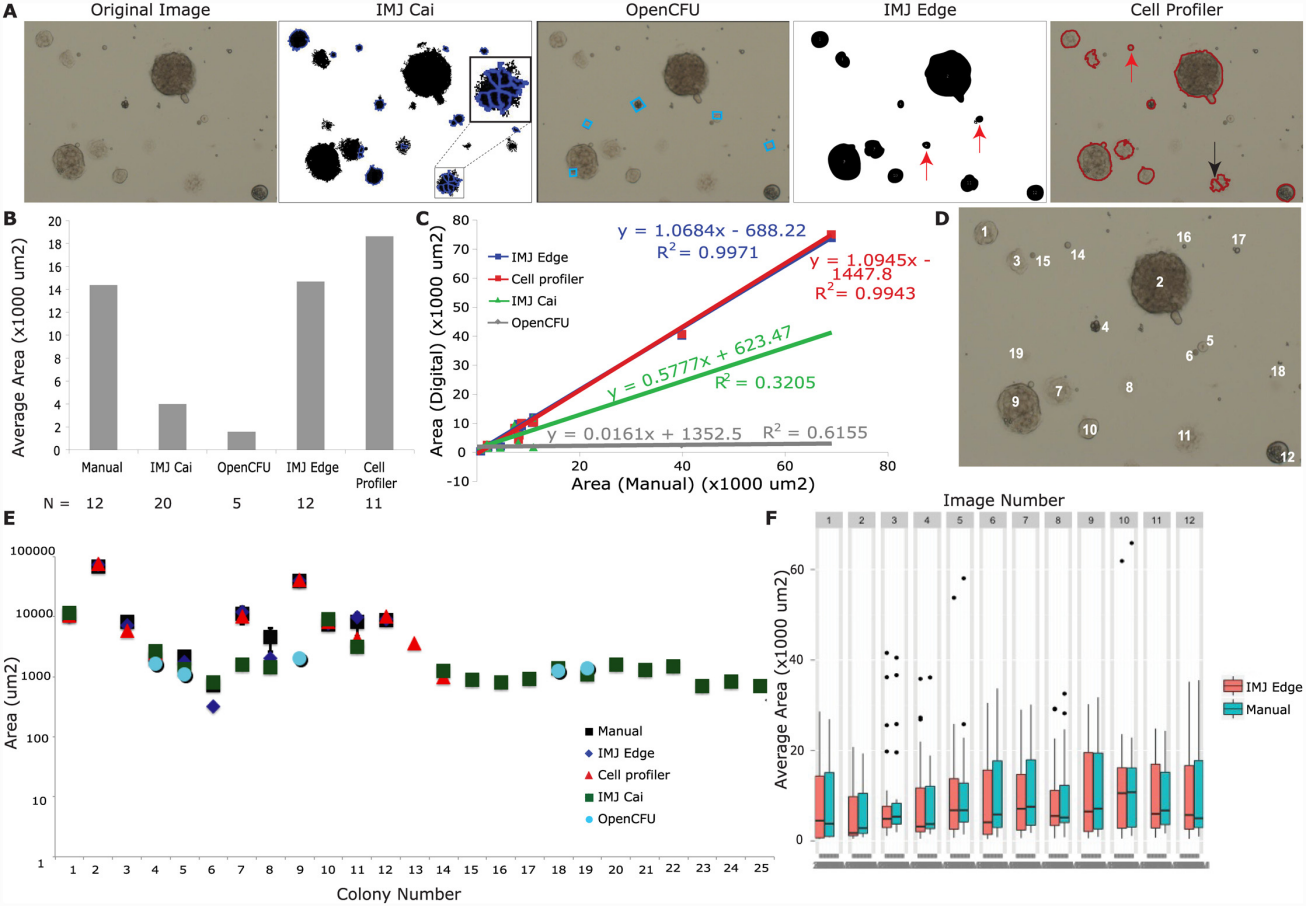
Comparison of different digital colony detection methods with manual measurements for tumorspheres. The first image in (A) is the original image. The rest are processed images from the colony detection methods. IMJ Cai has the thresholded areas in black, while the detected colonies have a blue outline around them. The inset shows a magnified view of colony that was segmented into 7 parts. Open CFU also has blue bounding boxes around the detected colonies, while Cell Profiler has red outlines. IMJ Edge has the detected colonies in black. (B) shows a plot of average area measured by the different detection methods. The number of colonies detected is shown is given by N below each method. (C) compares the average area (of 5 measurements) of each individual colony from digital methods (y-axis) with manual measurements (x-axis). The equation of the linear least-squared fit and goodness of fit R2 is given for each method. (D) labels each colony with its respective number. (E) plots the average area for each colony (labeled in D) against the colony number. Error bars are standard errors from 5 measurements. (F) is a boxplot showing the measurements of tumorspheres from 12 images measured manually and using IMJEdge.

First, I tested the Cai IMJ macro. Parameters such as minimum and maximum colony size were measured manually on either ImageJ or Adobe Photoshop. In the macro (IMJ Cai) the following parameters were designated: Background (radius = 50), set Threshold (0, 234), Analyze particles (Size: 700-Infinity, Circularity: 0.3–1). Fig 2A shows that following thresholding, colonies appear black on a white background. However, due to differences in opacity, colonies were segmented into several smaller pieces (inset in Fig 2A IMJCai), leading to an overestimation of the number of colonies. Furthermore, several colonies were not counted due to filtration of particles with circularity less than 0.3. The measured particles are indicated by a blue outline. IMJ Cai yielded 20 colonies, mostly counting segments of large colonies and some small colonies, with an average colony area of 4000 um^2^ (Fig 2B). This is in contrast to the area measured by manual counts- 14,379 um^2^ (Fig 2B). To compare the correlation between digital and manual measurements, the individual areas of digital counts were plotted against averaged manual measurements (Fig 2C). The linear least-squares fit of IMJ Cai measurements resulted in a line with slope 0.57 and R^2^ value of 0.32, suggesting that they did not correlate well with manual measurements. This is further shown by an analysis of the average areas of individual colonies (Fig 2E).

Colonies detected by various methods were matched against each other, and unique colonies (not measured in the manual method) were assigned additional colony numbers (Fig 2D). For example, Colony 11 in Fig 2E was segmented into two parts by CellProfiler (black arrow, Fig 2A): the larger part was matched to manual Colony 11, and the smaller part was counted as Colony 13. Similarly IMJCai segemented Colony 11 into 7 parts (inset, Fig 2A): the largest part was matched with Colony 11, and the rest were given additional colony numbers. To avoid confusion, all the additional colony numbers are not shown in Fig 2D. To demonstrate the difference in measurement methods, the average areas and the standard errors of individual colonies are plotted on the graph in Fig 2E. As expected, the standard errors in the automated methods were zero, hence error bars are shown only for manual measurements. As shown in Fig 2E, the green squares (IMJ Cai) deviate markedly from the black squares (Manual measurements), especially for Colonies 7, 8 and 11 (Colony numbers in Fig 2D). The additional colonies detected by IMJCai, including the large colonies segmented into parts, form the trailing line of colonies 14–25 in Fig 2E. Altogether, this analysis shows that despite manual optimization of threshold values, IMJCai did not yield accurate colony counts.

Next, I analyzed the images with OpenCFU, which failed to detect large colonies (Fig 2A), even though the minimum colony size was set to 1000 um^2^ and the maximum colony size was set as infinity. The colonies that were detected are shown in blue boxes in Fig 2A. OpenCFU was able to detect colonies in the size range of 1000–2000 um^2^, for example, Colony 4 and Colony 5 (Colony numbers in Fig 2D). It only detected a part of colony 9 (size 1996 um^2^), while manual estimation of full colony size was 39, 884 um^2^. OpenCFU’s limitation was not restricted to size, but also included detection. Several colonies in the size range of 1000–2000 um^2^, such as Colonies 14–17, were not detected. OpenCFU detected only 5 colonies, two of which were excluded in manual analysis, as they are too small. Due to these reasons the average colony size measured by OpenCFU was only 1589 um^2^ compared to 14,379 um^2^ by manual estimation (Fig 2B). The linear least squares fit of individual colony areas resulted in a flat line with slope 0.016 (Fig 2C). Fig 2E shows that OpenCFU area measurements of Colony 4 and 5 are close to manual measurements. However, OpenCFU measures only a part of Colony 9, and also measures Colony 18 and 19, which were not selected for manual measurement due to small size. Altogether, my data demonstrates that OpenCFU does not detect all colonies and does not significantly correlate with manual measurements.

Finally, I tested IMJ Edge macro and the Cell Profiler pipeline, and found that they detect colonies more accurately. IMJ Edge yielded values closest to manual measurements, for all characteristsics that were analyzed. The following parameters were designated: Background (radius = 80), Gaussian Blur (sigma = 2), Maximum (radius = 2), Minimum (radius = 3), Remove outliers (radius = 12), Analyze particles (Size: 50-Infinity, Circularity: 0.2–1). Twelve colonies were detected with an average area of 14674 um^2^, compared to the manual estimation of 14378 um^2^ (Fig 2B). Comparison of individual colony measurements generated a very good correlation. The linear least squares fit yielded a line with slope 1.06 and R^2^ value 0.9971 (Fig 2C), suggesting that IMJ Edge correlates extremely well with manual measurements. This can be further visualized in Fig 2E, where IMJ Edge measurements (blue rhomboids) match the best with manual measurements (black squares), as both measure the same 12 colonies with small deviations.

To analyze tumorsphere images on CellProfiler, they were first inverted prior to Background subtraction, because tumorspheres appear dark on a light background, while the module is optimized for white colonies on a dark background. The following parameters were used: Identify Primary Objects (typical diameter 13–200 as measured on Adobe Photoshop), Otsu thresholding, Filter Object (minimum form factor = 0.2, minimum radius = 8). The minimum radius for filtering is different in CellProfiler because this value is defined as pixels, as opposed to IMJ Edge where it is scaled from pixels to micrometers. Despite good correlation, there are some differences in the results obtained from CellProfiler when compared with IMJ Edge and manual measurements. For example CellProfiler detected Colony 14 (red arrow in Fig 2A, Cell Profiler image), but not Colony 5 and 8 (red arrows in Fig 2A, IMJ edge image), both of which are bigger than Colony 14. In addition, CellProfiler detected Colony 11 as two colonies (black arrow, Fig 2A). Furthermore, CellProfiler does not detect the boundaries of colonies well, yielding fuzzy and noisy edges. Measurements of colony number and average area deviated slightly from manual measurements (11 colonies compared to 12; and 18,629 um^2^ compared to 14,378um^2^) (Fig 2B). Encouragingly, least squares fit of individual areas resulted in a straight line with slope 1.09 and R^2^ value 0.9943 (Fig 2C). This demonstrates that Cell Profiler correlates significantly with manual measurements. Since Cell Profiler did not detect Colonies 5, 6 and 8, but detected Colonies 13 and 14, the plot of individual areas shows CellProfiler measurements are very similar to manual measurements except at those points (Fig 2E).

To test the high-throughput processing ability of IMJEdge, a folder of 12 images was analyzed. While these images are from the same experiment, and were taken with the same microscope settings, they vary in the number and size of tumorspheres, artefacts, illumination etc. Manual measurements were taken twice, averaged and compared to IMJEdge measurements in the boxplot in Fig 2F. The median, 25^th^ and 75^th^ quartile values from the two methods are close, suggesting that IMJEdge yields measurements close to manual measurements. Notably manual measurements of 12 images took 4 hours, while IMJEdge analyzed the 12 images in under 2 minutes.

Overall, both the IMJ Edge and Cell Profiler pipeline have the advantage of being simple, automated, and more reproducible than manual measurements. Among all methods that rely on thresholding, Cell Profiler yielded the most accurate results. However, IMJ Edge was faster (20 seconds compared to 2.5 minutes on Cell Profiler) and more accurate than all other methods that were tested in this study.

### Bacterial/Yeast colonies

Counting cell colonies is a standard method for determining the number of colony forming units (CFUs) in microbiology and immunology. For example, counting of bacterial and yeast colonies is used for precise, quantitative assessment of pathogens in clinical samples and for establishing the efficacy of vaccines. Current methods rely on an investigator manually scoring colonies, color and type, a method that is slow, tedious, and has low reproducibility.

**Fig 3:**
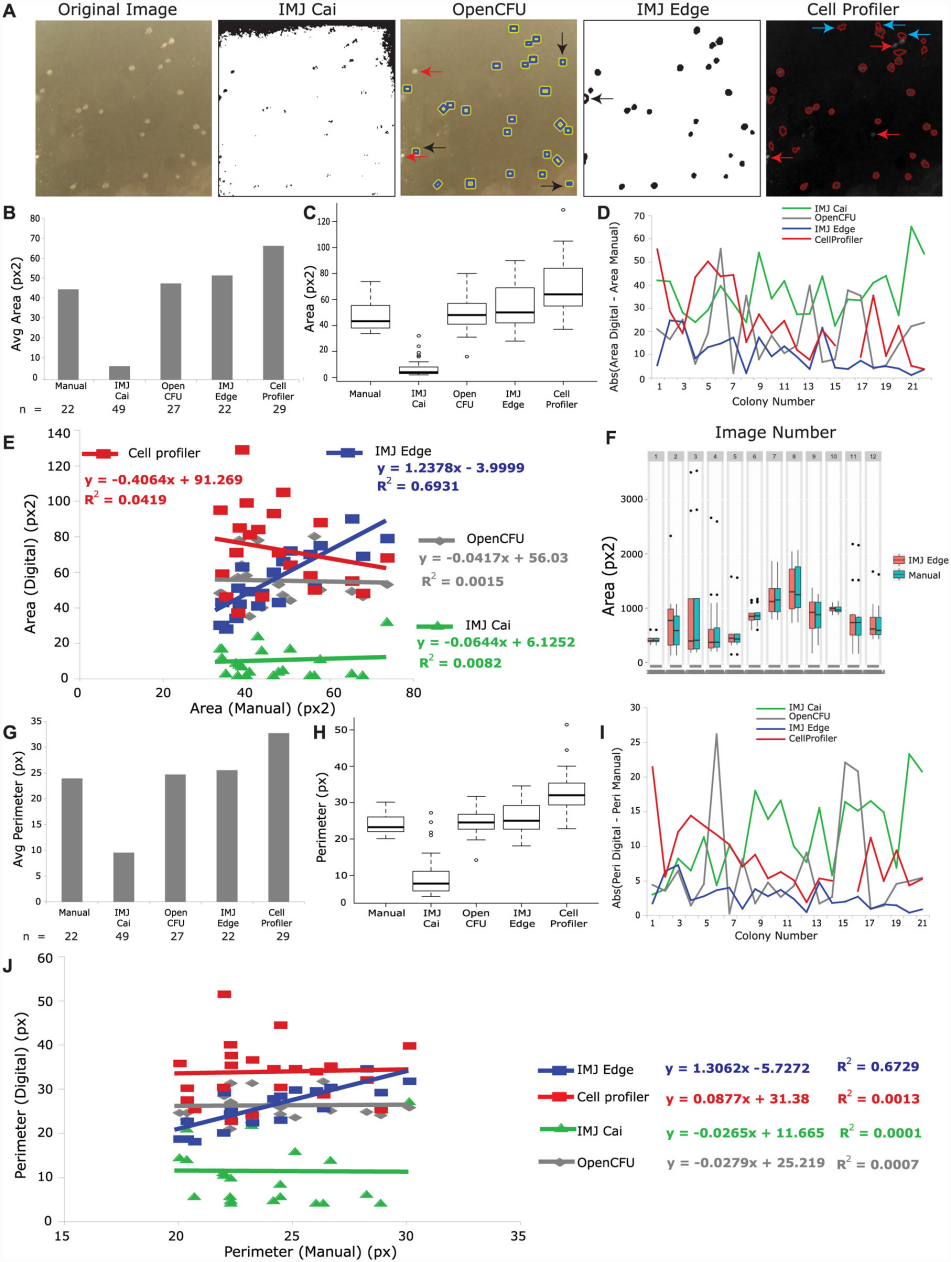
Comparison of digital methods for colony detection for bacterial colonies. (A) shows the original image, and masks/overlays of detected colonies by various methods. (B) Plot of the average of area measurements, while (C) is boxplot showing the distribution of area measurements by various methods. (D) is a plot of the absolute values of difference between digital and manual measurements of each colony. (E) plots the digital measurements of Area against the manual measurements. The equations of the least square fit line is given with R2 value. (F) displays the distribution of bacterial colony area measurements from 12 images by manual and IMJEdge methods. (G) is a plot of average perimeter, and (H) is a boxplot of perimeters measured by different methods. (I) plots the absolute deviations of different methods from manual measurements of perimeter for each colony. (J) plots the digital measurements of Perimeter against the manual measurements.

While thresholding for IMJ Cai, a balance was maintained between the setting edges and colonies as background. The following parameters were assigned: Background (radius = 100), set Threshold (216, 247), Analyze particles (Size: 2-Infinity, Circularity: 0.3–1). Fig 3A shows that while the colonies appear black, the top edge was not divided into background completely. To address this issue, I first increased the minimum threshold value, but this led to elimination of some colonies as well. Consequently, the sizes of detected colonies were estimated to be smaller than their actual sizes. In addition, several dots from the edges were also detected as colonies. Hence the number of colonies detected by IMJ Cai was greater than by manual measurements (49 *versus* 22; Fig 3B). Further, both the average area (Fig 3B) and the distribution of the areas (Fig 3C) of detected colonies were lower than manual measurements (6.9 *versus* 46.7 sq px). When absolute values of deviation from manual measurements were plotted for individual colonies (Fig 3D), IMJ Cai deviated the most from manual measurements compared to other methods. To determine the correlation between digital and manual measurements, the individual areas obtained from digital analysis were plotted against averaged manual measurements (Fig 3E). The linear least-squares fit of IMJ Cai measurements yielded a nearly horizontal line, suggesting that the measurements did not correlate. Similar comparisons were then made for colony perimeter. The average perimeter (Fig 3G) and the distribution of colony perimeters (Fig 3H) for IMJ Cai was lower than the values obtained from manual analysis (9.5 *versus* 23.9px). In addition, the deviation of absolute perimeter values of individual colonies from manual measurements was the maximum, when compared with other quantification methods (Fig 3I). Similar to area, perimeter measurements on IMJ Cai also yielded a flat line when plotted against manual measurements (Fig 3J).

The following parameters were used for analysis by OpenCFU: Size (3–9999) and Inverted Threshold (0). OpenCFU counted bacterial colonies relatively well; it detected 19 of the 22 colonies measured manually. Only two colonies were undetected (indicated by red arrows in OpenCFU image in Fig 3A), and in some cases the space next to a colony was detected (marked by black arrows in Fig 3A). However, in spite of these differences, the number of colonies determined by this method was very similar to that obtained by manual measurements (27 *versus* 22). The average area (Fig 3B) and distribution of areas (Fig 3C) determined by OpenCFU was slightly higher, but similar to manual measurements. Similarity in colony number, average colony area and area distribution suggested that OpenCFU measurements might correlate well with manual measurements. However, the linear least-squares fit of OpenCFU measurements plotted against manual measurements resulted in a line with a negative slope of 0.41, suggesting poor correlation. This might be caused due to square colony selection areas by OpenCFU compared to elliptical/circular selection areas used in manual analysis. Similar to area, average perimeter (Fig 3G) and distribution of perimeters (Fig 3H) were slightly higher than manual measurements. The individual colony perimeters determined by OpenCFU deviated only slightly from manual perimeter values for most colonies (Fig 3I). In spite of close perimeter distributions, the values did not correlate well with manual perimeter measurements, yielding a linear least-squares fit line with slope negative 0.028.

Next, I tested IMJ Edge, and found that it detected colonies accurately and correlated well with manual measurements. In IMJ Edge, background subtraction was substituted with a step to eliminate scratches on the plate (remove outliers). Since these scratches showed as white, the color of remove outliers 1 was chosen as white. The following parameters were used: Gaussian Blur (sigma = 1), Remove outliers 1 (radius = 2, color = Bright), Analyze particles (Size: 2-Infinity, Circularity: 0.2–1). IMJ Edge detected all visible colonies with a filling error in one (black arrow, IMJ Edge; Fig 3A). This colony was probably not filled completely due to its location near the edge of the image. Both the average area (Fig 3B) and the distribution of areas (Fig 3C) was slightly higher than manual measurements, but the deviation of individual colonies from manual measurements (Fig 3D) was the least when compared to all other methods of analysis. Importantly, IMJ Edge measurements displayed the best correlation with manual measurements (Fig 3E), with the least squares fit giving a slope of 1.237. Testing a folder of images with IMJEdge demonstrated that the same parameters yield measurements very close to manual measurements (Fig 3F). Besides the median, 25^th^ and 75^th^ percentile values, even the outliers are close between IMJEdge and Manual method for most of the images. Similarly, perimeter measurements on IMJ Edge, including average perimeter (Fig 3G) and distribution (Fig 3H), were closest to the values obtained by manual analysis, in comparison to all the other digital quantification methods. The absolute deviation of individual colonies from manual measurements was also the least, when compared to other methods (Fig 3I). As expected, the perimeter values on IMJ Edge showed significant correlation with manual measurements (Fig 3J).

Finally, I tested the accuracy of CellProfiler in quantifying the characteristics of bacterial colonies. Although this method detected colonies relatively well, it was not accurate. The following parameters were used for analysis: Identify Primary Objects (typical diameter 2–20), Otsu thresholding, Filter Object (minimum form factor = 0.2, minimum radius = 3). Although manual measurements determined the minimum radius as two, three was used in this analysis because a radius of two yielded 87 colonies (3 -fold higher than the number of colonies determined by manual measurement). Under these settings, CellProfiler detected most but not all colonies (red arrows, Fig 3A-Cell Profiler). Some additional features on the image were also detected as colonies (blue arrows). As a result of these false positives and false negatives, CellProfiler detected 29 colonies, compared to 22 from visual estimation. In spite of false positives, the average area and area distribution are higher on CellProfiler compared to manual analysis. When CellProfiler values were plotted against those obtained by manual measurement, I obtained a line with negative slope, suggesting poor correlation. Similar to area, CellProfiler also yielded higher perimeter values (average and distribution) than manual measurements (Fig 3F and 3G). Furthermore, individual colony areas and perimeters measured on CellProfiler varied significantly from manual analysis (Fig 3D and 3H). Similar to area, these perimeter values also failed to correlate with manual measurements (Slope = 0.08, Fig 3I).

In summary, IMJ Edge outperformed all other image analysis methods tested for colony detection and yielded the most accurate results, closest to those obtained from manual measurements. A possible reason for slopes of 1.3 in the least squares fit of area and perimeter could be variability in manual measurements. Since the size of bacterial colonies is small, a small error in measurement could introduce a large percentage error. The variability in manual measurements are indeed quite large, and are shown in S2 Fig. This problem can be circumvented by automation.

### Clonogenic assays for tumor cells

Clonogenic assays are used to study the effectiveness of anti-cancer agents such as drugs and radiation[23, 25, 26]. Specifically, cancer cells are allowed to grow in the presence of the agent, which is followed by an estimation of cell survival and proliferation by counting the number of stained colonies containing more than 50 cells. Since manual counting is time-consuming and subjective, I tested the various digital image analysis methods for their accuracy and reliability in counting the number of colonies in a clonogenic assay.

**Fig 4:**
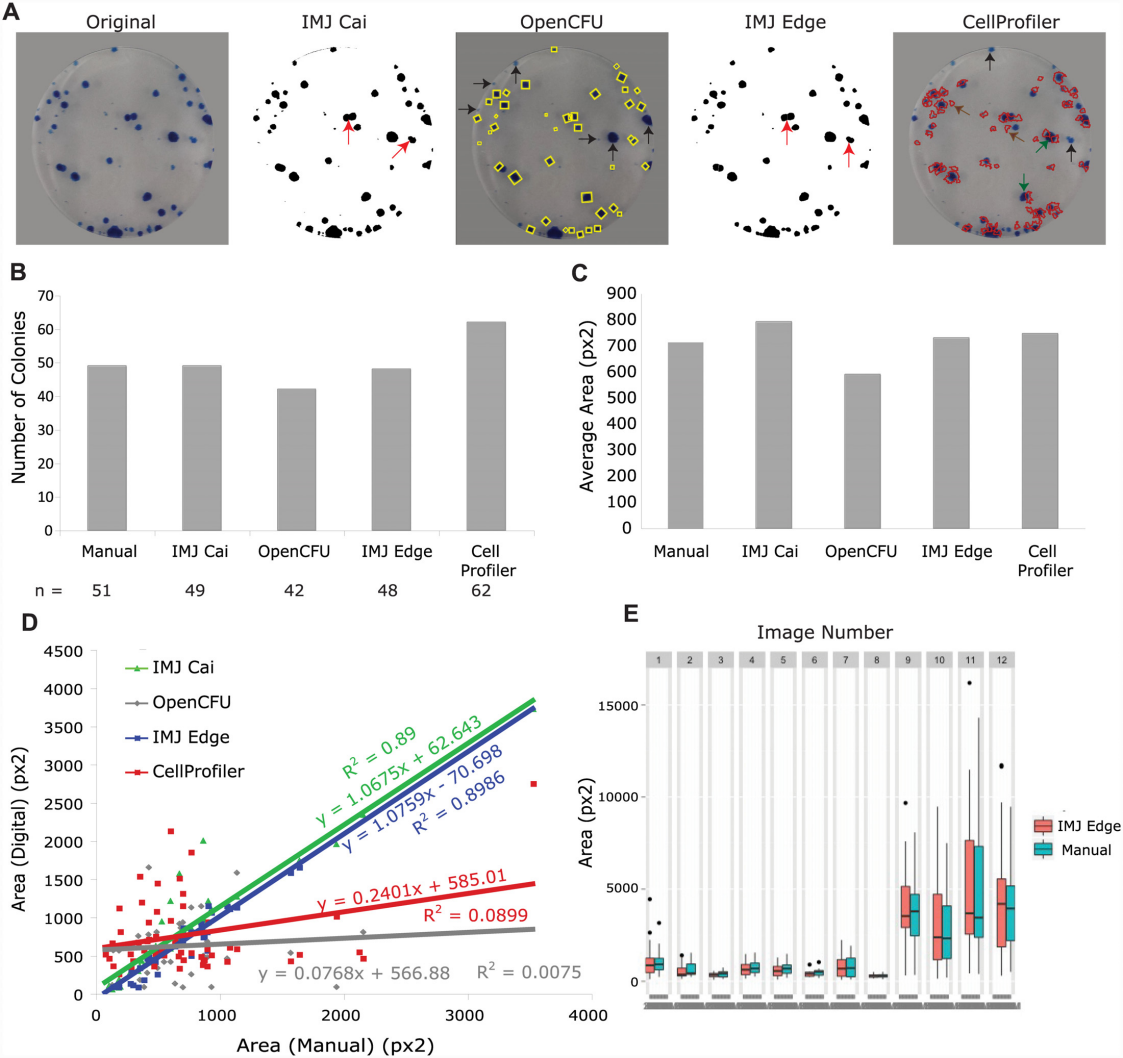
Comparison of digital methods for colony detection for bacterial colonies. (A) shows the original image, and masks/overlays of detected colonies by various methods. Red arrows in IMJ Cai and IMJ Edge mark unsegmented colonies. Black arrows mark undetected colonies. (B) Plot of the number of detected colonies, and (C) is a plot of the average colony areas. (D) plots the digital measurements of Area against the manual measurements. The equations of the least square fit line is given with R2 value. (E) is a boxplot of measurements from 12 Clonogenic assay images by manual and IMJEdge methods

First, I tested the Cai ImageJ macro (IMJ Cai) using the following parameters: Background (rolling = 50), set Threshold (0–199), Analyze particles (Size: 50-Infinity, Circularity: 0.2–1). As shown in Fig 4A, although colonies were detected accurately, some were improperly segmented (red arrows). As a result, analysis by IMJ Cai yielded 49 colonies with an average area of 876 px^2^, compared to 51 colonies and 745 px^2^ determined by manual measurement (Fig 4B and 4C). Area measurements by IMJ Cai correlated well with manual measurements (Fig 4D). The linear least-squares fit of IMJ Cai measurements generated a straight line with slope 1.06 and R^2^ value 0.89.

OpenCFU did not detect clonogenic colonies accurately. The parameters used for OpenCFU analysis were: Size (5–999) and Threshold (15). Although this resulted in the detection of colonies in the median area range, small (horizontal arrows) and large (vertical arrows) colonies were missed (Fig 4A). As a result, OpenCFU only detected 42 colonies, compared to 51 by manual measurement (Fig 4B). Further, the colony areas measured by OpenCFU did not correlate with manual measurements (Fig 4D), resulting in a nearly horizontal linear least squares line (slope = 0.076).

Analysis of clonogenic colonies with IMJ Edge was done using the following parameters: Subtract Background (radius = 50), Gaussian Blur (sigma = 0.5), Remove outliers (radius = 2), Maximum (1), Minimum (4), Remove outliers (5), Analyze particles (Size: 50-Infinity, Circularity: 0.2–1). Several denoising and ‘Remove outliers’ steps were inserted to deselect the pixels at the edge of the plate (S1 Appendix). Under these conditions, IMJ Edge yielded an almost accurate number of colonies (49 *versus* 51 from manual measurement). The lower value is likely due to inaccurate segmentation (red arrows–Fig 4A). In addition, area measurements also correlated extremely well with manually obtained values (Fig 4D), yielding a linear least squares line of slope 1.076 and R^2^ value of 0.89. Again, a folder of 12 images from the same experiment were analyzed and compared to manual measurements (Fig 2E). Parameters were chosen such that most images gave accurate results. Since parameters were not optimized for individual images, IMJ Edge yields some outliers (Image 2, 9, 11, 12 in Fig 2E). However, the median, 25^th^ and 75^th^ quartile values from the two methods were very close for all 12 images, suggesting that IMJEdge measurements are close estimates of manual measurements.

In contrast, CellProfiler did not detect clonogenic colonies accurately. The parameters used were: Identify Primary Objects (typical diameter 5–35), Otsu thresholding, Filter Object (minimum form factor = 0.2, minimum radius = 5). The method to distinguish clusters was tested for both intensity and shape, yielding similarly inaccurate detection of colonies (Fig 4A). Several colonies were not detected (black arrows), some colonies were segmented incorrectly and only some portions were measured (green arrows), and finally, some areas that did not contain any colonies were falsely measured (brown arrows) (Fig 4A). As a result, CellProfiler detected 62 colonies instead of 51 (manual). Surprisingly, in spite of inaccurate colony detection, the average colony area was the same as estimated from manual measurements (746 sq px and 745 sq px respectively). As expected, the individual areas did not correlate with manual measurements, and the linear least squares fit line had a slope of 0.24.

Altogether, for this high contrast image, IMJ Edge and IMJ Cai performed equally well at detecting and measuring colonies. Although they both had minor problems with correct segmentation of merged colonies, they correlated extremely well with manual measurements.

### Cell detection and measurement

**Fig 5:**
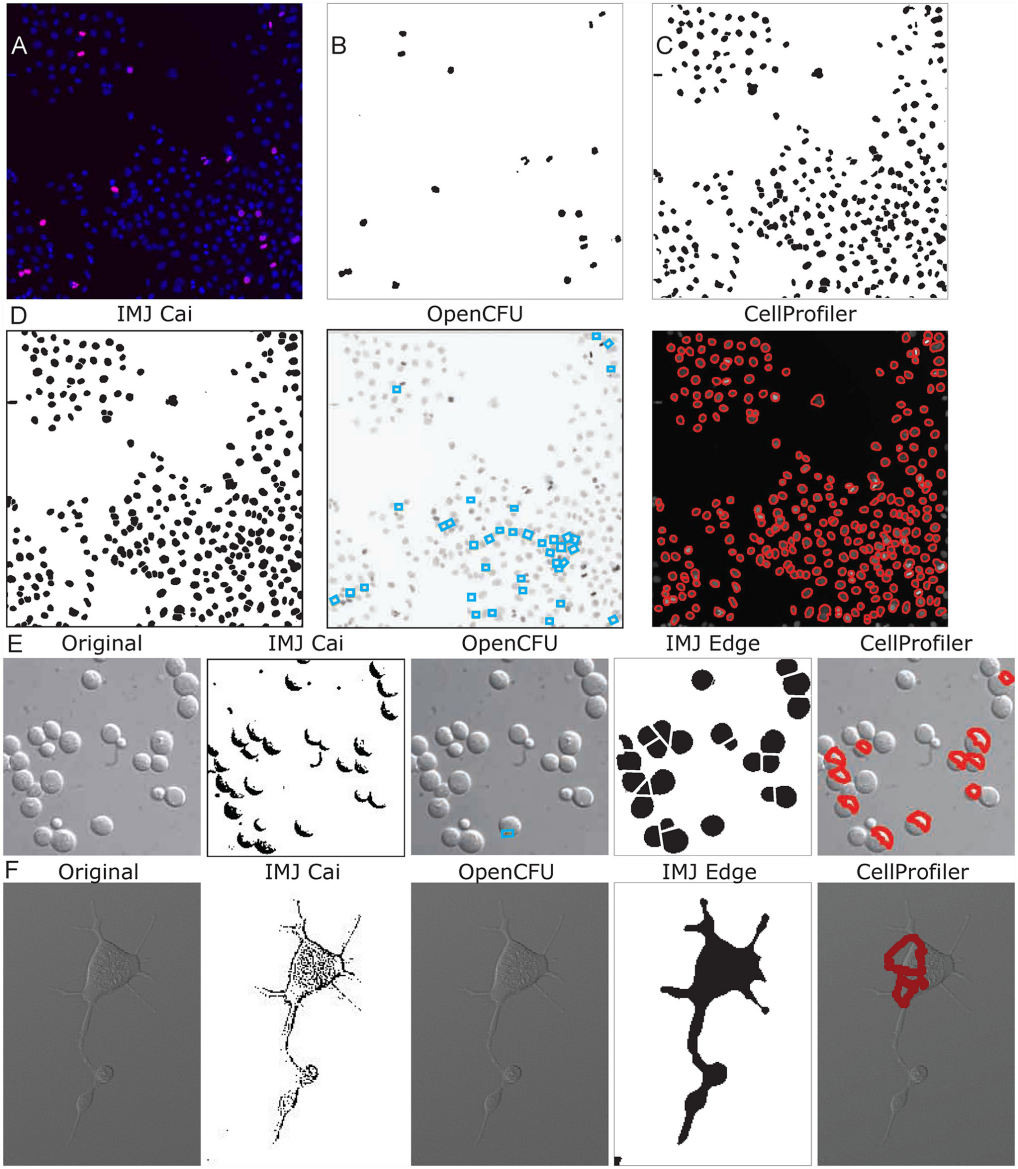
Application of IMJ Edge for detection of cells. (A) RGB image showing DAPI staining of nuclei in blue, and phospho-histone3 staining in red. (B) shows the red channel processed with IMJ Edge to calculate the number of dividing cells. (C) shows the blue channel processed by IMJ Edge to identify all the nuclei. (D) shows the blue channel of (A) processed by IMJ Cai, OpenCFU and Cell Profiler. The detected nuclei are outlined in blue boxes by OpenCFU, and in red circles by CellProfiler. (E) shows the original Nomarski image of yeast cells, and the processed images by various methods next to it. (F) shows the Normarski image of a 293 cell and its processed versions next to it. The segmentation step was skipped in IMJ Edge to prevent segmentation of cell processes.

In addition to colony detection, IMJ Edge can also be used to detect cells from both fluorescent and brightfield images. Images can be processed to measure differences in cell count, intensity, or morphology, according to the application. Fig 5A shows a fluorescent image of human HT29 cells downloaded from the CellProfiler website (Example Human Cells under basic pipelines). The nuclei are stained with DAPI (blue), and the dividing cells with an antibody against phospho-histone3 (red). The channels can be separated in ImageJ using the command- Image-> Color-> Split channels. The red and and blue channels were processed separately by IMJ Edge to obtain the number of dividing cells (Fig 5B), and the total number of cells (Fig 5C), respectively. In this case, IMJ Edge could be applied to test drug efficacy or the effect of genetic manipulations that affect cell division. This assay can also be applied to determine changes in protein expression, by using the outline of cells to measure the intensity of immunostaining in a particular channel. For such fluorescent images, thresholding methods IMJ Cai and CellProfiler detected cells equally well, while OpenCFU did not (Fig 5D).

IMJ Edge can also detect and measure cells in Nomarski images. Fig 5E is an image of yeast cells (courtesy: [49]), and its processed forms by digital methods. IMJ Edge was fairly accurate in detecting cells, though the cell areas are slightly larger than manual measurements (data not shown). The larger areas are probably caused by inclusion of areas in between cells in a cluster. However, the other methods only detected portions of some cells. IMJ Edge can also detect non-circular cells as shown in Fig 5F. While IMJ Cai and OpenCFU fail to detect the cell as an object altogether, CellProfiler detected cell segments as separate cells. IMJ Edge detected the cell accurately when the segmentation step was skipped (to prevent segmentation of the cell processes as separate particles). The ImageJ watershed segmentation algorithm does not function well for non-circular objects. Hence elongated cells like epithelial, muscle etc are not segmented well. This limitation of IMJ Edge can be overcome by seeding cells at low density, and having well-focused cell edges during image acquisition. While IMJ Cai and CellProfiler detect and quantify cells accurately in fluorescent images well, they fail to do so in Nomarski images.

### Tissues and Cellular Assays

**Fig 6:**
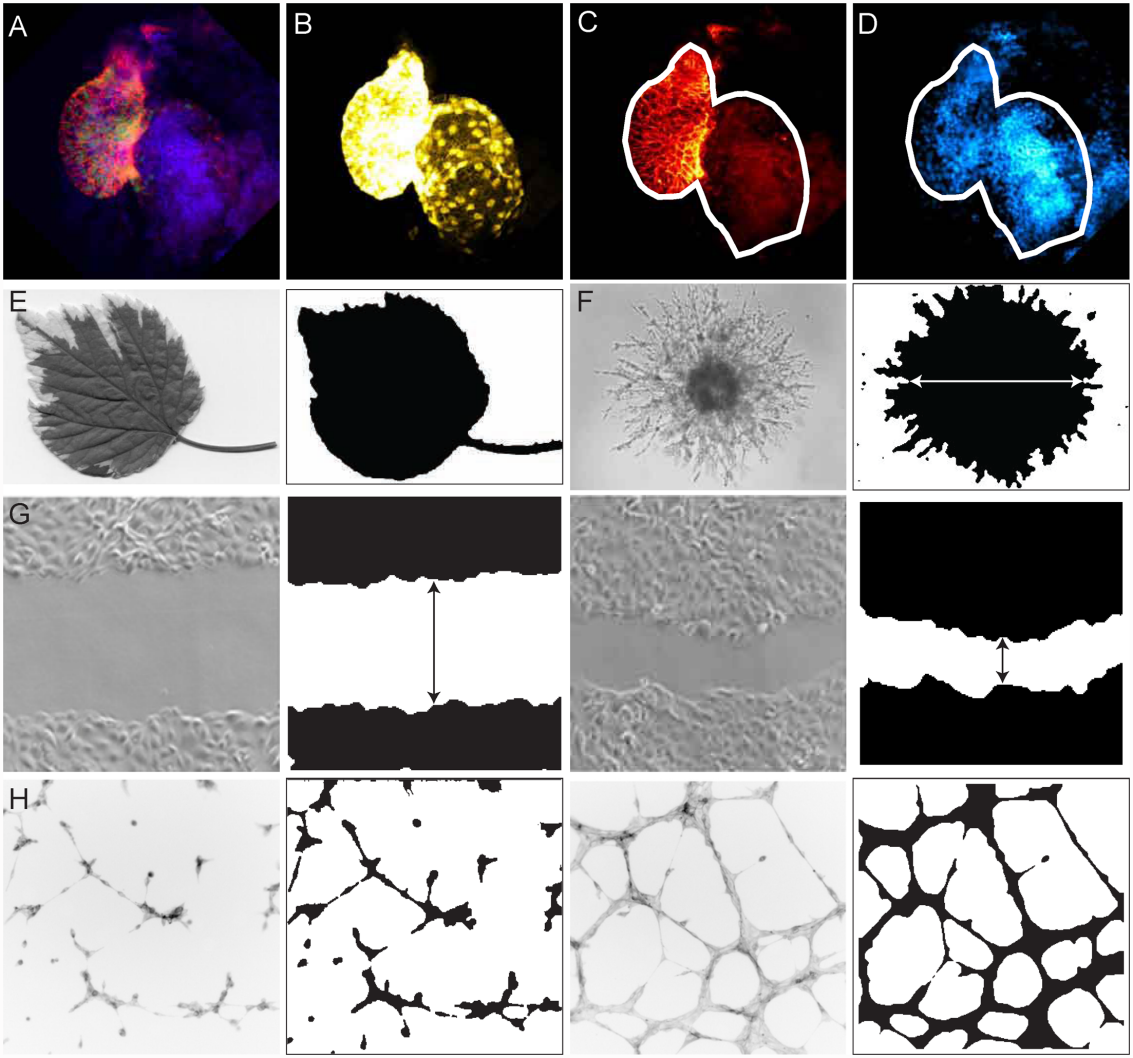
Application of IMJ Edge on tissue images and cellular assays. (A) Image of a stained zebrafish heart with heart cells in green (*cmcl2*:*gfp* line), nuclei in blue (DAPI), and heart cell membranes stained in red (Alcama antibody). The green channel (B) was processed by IMJ Edge, and the outline overlaid on the red (C) and blue (D) channels (white outline). (C) and (D) show that there are stained cells outside of the outline that will be measured if outlines are not overlaid. (E) Picture of a leaf processed by IMJ Edge. (G) Two time points of a scratch assay processed by IMJ Edge. Black arrow marks the difference between the edges of epithelial cells. (F) Picture of an invasion assay from spheroid processed by IMJ Edge. The white arrow shows the diameter. (H) Images of tube formation assay for endothelial cells processed by IMJ Edge. The fill step was skipped to avoid filling in the areas between tubes.

Thus far, I have tested the utility of IMJ Edge in detecting individual cells or colony forming units. However IMJ Edge can also be applied to other cellular assays, as well as analysis of tissues and organs. Fig 6A shows a zebrafish heart isolated from the transgenic line *Tg (cmlc2*:*EGFP)*, that expresses green fluorescent protein (GFP) in cardiomyocytes [4]. Cells from the pericardium and other surrounding tissues are also attached to the heart and can be visualized by staining with DAPI (blue) and Alcama antibody (red). To count and measure expression specifically in cardiomyocytes, the channels were first split, and the green channel (Fig 6B) was processed by IMJ Edge to obtain the boundaries of the heart. The outline was then superimposed on the red (Fig 6C) and blue (Fig 6D) channels to reveal the included and excluded regions. Fig 6C and 6D show red and blue staining outside the white outline that would have skewed the results without the exclusion step. This outline was then used to specify the ROI in ImageJ, and measurements of protein expression (Alcama staining in red) and cell numbers (DAPI in blue) were made specifically for cardiomyocytes only (data not shown). This technique can also be used for detecting and measuring specific cell types in various tissues. For example, tissue sections can be stained with cell-specific marker and BrdU to identify proliferating cells of that type specifically. Another example is shown in Fig 6E, an image of a leaf in ImageJ. The edges of the leaf are not green, and hence thresholding methods like IMJ Cai do not detect the entire leaf (data not shown). IMJ Edge however detects both the white edges and the green edges, and since the interior is filled during the macro, the entire leaf is detected.

In addition to tissues, IMJ Edge has utilities in several cellular assays too. Shown in Fig 6F is the image of an invasion assay of tumor spheroids (courtesy: Trevigen.com). IMJ Edge could detect the edges and fill up the interior, making it easier to measure the diameter of the invasive area. IMJ Edge was also tested for its utility in scratch and tube-formation assays. Shown in Fig 6G are images of a scratch assay (courtesy: [50]), processed with IMJ Edge. IMJ Edge highlighted the edges of the scratch at the start of the assay, and at later time points, it successfully detected the leading edge of migrating cells. Finally, IMJ Edge was used to process images of a tube formation assay (courtesy: [51]). IMJ Edge correctly identified the edges of tubes, and enabled reliable measurement of the area covered. Note that in this assay, the ‘fill’ step was skipped to avoid filling the areas between tubes. Importantly, in all these assays, once the parameters of IMJ Edge were specified manually for an individual image, the analysis could be automated and extended to an entire folder of images.

## Discussion

In the present study, a new IMJ Macro based on edge detection, and a new CellProfiler pipeline have been presented, tested and compared to two alternative digital methods for cell and colony quantitation. Several alternative digital methods were excluded from this study due to technical issues or inconsistent results. For example, Brugger et al created a standalone application in MATLAB, that can function without a complete installation of MATLAB[52]. However, the method is optimized for blood agar plates, and requires images procured under specific conditions such as blue dark illumination. The ImageJ plugin by Sieuwerts et al failed to function properly, and consistently displayed the following error message: ‘the next step requires a binary image’[53]. On manual conversion to ‘binary’, the plugin could be executed to completion. However, this method is not compatible with automation of multiple images because it requires manual intervention at several steps including thresholding. Furthermore, two steps of image inversion result in loss of resolution and incorrect colony detection, despite accurate thresholding (Figure A in S3 Fig). Consequently, several colonies remained undetected (vertical arrows), while some were incorrectly segmented and measured as separate colonies (horizontal arrows). Another option is Clonocounter, a program written in Java and available freely [54]. However, this method is inflexible, does not correct for non-uniformity in illumination or scanning, and is non-tunable by the user. An example of this method’s use is shown in Figure B in S3 Fig. Here, only the small green areas were detected while excluding full colonies. In addition, this program is optimized exclusively for circular selected areas on 6-well plates. The user is prompted to pick a circular area in the image provided, but the maximum size of the circular area is limited. Therefore, depending on the image size and resolution, this circular area may include only a couple of colonies. Consequently, the entire image could not be analyzed, and this method could not be compared to others. Guzman et al [55] published an ImageJ plugin ColonyArea, for quantitating clonogenic assays. The method has useful features like automated detection, separation and thresholding of individual wells from a multiwall plate. However, the program only works for multiwell plates and could not be used to analyze images lacking well boundaries. Furthermore, it measures the colony area as a percentage of the well area, and does not calculate the number of colonies, or their individual areas.

This is not the first manuscript using edge detection for cellular assays. Treloar and Simpson use ImageJ and MATLAB edge detection methods to detect the leading edge in cell migration assays [56]. The ImageJ method consists of two steps of Edge Detection, with a Sharpening and Thresholding step applied in between. Finally, they used the ImageJ wand tracing tool to manually select the leading edge of each cell migration assay. IMJEdge differs from this method because it has several additional steps for background subtraction, smoothing, removing outliers, filling the detected colonies, filling additional colonies with gaps in edges, segmenting colonies, measuring all objects in one step with Analyze Particles (instead of manually with the wand tool), and the ability for automation. Furthermore, despite the presence of two common steps (edge detection and sharpening), the order of these steps is different yielding different processed images (Figure D in S3 Fig). While Treloar and Simpson’s ImageJ method is good for detecting the leading edge of biological objects, the method does not fill in and automatically measure all the objects in an image.

Among the methods tested, IMJ Cai thresholding method worked best when the imaged cells and/or colonies possessed very good contrast compared to background. This included the clonogenic assay and fluorescent staining. It failed to accurately analyze brighfield/Nomarski images of unstained cells and colonies. The software OpenCFU is also based on thresholding, with additional steps to increase robustness by eliminating the edges of plates and flasks. To do this, OpenCFU tests all possible values of threshold on the given image, and quantifies only those areas that consistently emerge as morphologically valid regions. However, my results show that OpenCFU eliminated several colonies, especially the larger ones (Figs 2A and 4A). Among the thresholding methods tested here, CellProfiler generated the best results. It successfully detected colonies in low contrast images like those of tumorspheres and bacterial colonies, albeit with some errors and deviations from manual measurements. However, it also yielded very high errors in unevenly illuminated images, such as those of bacterial cell cultures and clonogenic assays. All these methods failed when the cells were non-circular, and the images lacked sufficient contrast (Fig 5F). Furthermore, none of these methods could be used for measuring tissues or cellular assays.

In summary, the currently available colony detection methods function adequately only when the cells and colonies have good contrast compared to background, when the objects are large, circular and well spaced, and when the images are evenly illuminated. When these optimal conditions are violated, most colony detection methods lose accuracy. In contrast, IMJ Edge detects cells and colonies accurately in all conditions tested, including low-contrast, uneven illumination, and non-circular cells. IMJ Edge could also be used for measuring cellular assays, and tissue morphology. It also displayed the highest correlation with manual measurements, and was 10-fold faster than CellProfiler (~10s compared to ~2 minutes/image), and 60-fold faster than manual measurements (~10s/image compared to an average of ~10 minutes/image). My data demonstrates that IMJ Edge is superior to all other image analysis methods tested in this study, in speed, accuracy, reliability and versatility.

In spite of its accuracy and versatility, IMJ Edge has some limitations. Edge detection operates by detecting sudden changes in grayscale values, making them susceptible to counting the edges of plates and miscounting merged colonies. Counting of plate edges is avoided by manual cropping of images before processing. Alternatively, a template mask can be used (for example a circle for plates), and aligned computationally with the actual image[57]. Miscounting of merged colonies can be alleviated by the segmentation step, but probably not overcome completely. Notably, the segmentation algorithm can be applied reliably only to circular/elliptical regions, and should not be used for detection of non-circular objects. Non-circular cells can be counted by IMJ Edge (without the segmentation step), by plating at low densities to obtain separated individual cells. Due to its dependence on detecting edges, this method will fail for extremely low resolution or unfocused images where the edges do not have a sudden change in greyscale values. Notably, accuracy of object detection is dependent on the parameters used. Hence, optimum parameters should be determined manually, using 4–8 images with varying number and size of cell/colonies. In addition, all the images in the folder should be in the same format (tiff, jpg etc), resolution and size, because the optimum parameters change with image quality, pixel density and compression.

In this study, IMJ Edge was used successfully to count cells and colonies accurately and reproducibly, under various imaging conditions. In addition, the ability to automate IMJ Edge makes it extremely compatible with high-throughput screens. There is a need for identifying new targets and drugs against cancer cells, as well as new antibiotics and vaccines against bacterial pathogens. Since IMJ Edge can accurately and automatically analyze MCTS, they can now be used in high-throughput drug screens. Such an approach will help yield better therapeutics since MCTS are shown to model chemotherapy resistance better than 2D cultures. Similarly automated analysis of the clonogenic assay will allow chemical and RNA interference high-throughput screens to identify new agents and targets for radiosensitization. Furthermore, IMJ Edge will reduce subjectivity, and save researchers innumerable hours. In summary, IMJ Edge is automated, fast, accurate, reproducible, and highly suitable for high-throughput screening.

## Supporting Information

S1 Appendix ImageJ Cell_Colony_Edge Macro. The macro given in text. The text in green explains the function of the following code. The parameters used for different images are also given.
(PDF)

S2 Appendix Customized versions of Cell_Colony_Edge macro for specific purposes. (PDF)

S3 Appendix Instructions for manual determination of parameters using ImageJ GUI. (PDF)

S4 Appendix Instructions for downloading and running the macro. (PDF)

S1 Fig Image Processing steps of IMJ Edge, and selection of parameters. (Figure A) Original Nomarksi image showing a single U266 cell (modified from [40]). The diameter was measured as 10 px in ImageJ. (Figure B) Image in (A) after background subtraction with different rolling ball radii (written in bottom right corner of each image). Rolling ball radius of 40 was selected (blue checkmark) because all edges were visible. (Figure C) Background subtracted image is then sharpened and enhanced (0.2%). This image is further processed by the “Find Edges” and “Make Binary” commands. (Figure D) The binary image is processed by the “Gaussian blur” command with different sigma radii (written in bottom right corner of each image), followed by filling and closing of holes. Red arrows show that debris is processed as an object when 0.25 or 1 is used as radius for Gaussian blur. Gaussian blur with radius 2 did not select debris as an object, and was selected (blue checkmark). (Figure E) Closed and Filled image in D is further processed by the “Maximum” command to increase size of each pixel. This brings the edges of gaps closer together (green arrows). Radius 2 was selected, as the edges of gap are close enough for filling and closing. (Figure F) Image in E was closed and filled. (Figure G) Size of pixels is reduced back. Radius 5 is chosen because size of selection is similar to the size of cell in original image. Overlay is shown for comparison purposes.
(TIFF)

S2 Fig Variability in Manual measurement of bacterial colonies. The plot is broken in two halves with different scales to represent different values on the y-axes with Colony numbers on the x-axis.
(TIF)

S3 Fig Other methods of colony counting not tested in paper. (Figure A) Original image of tumorspheres processed by different methods. (Figure B) Results of image processed by Sieuwerts et al ImageJ plugin. Vertical arrows point to undetected colonies, and horizontal to colonies that are only partly detected. (Figure C) Image of Clonocounter working on Image in Figure A. Only part of the image inside the circle is analyzed. In this part too, the green highlights show the detected colonies. The table underneath shows the results of clonocounter. (Figure D) Original tumorsphere image on the left followed by the image processed by Treloar and Simpson ImageJ method and by IMJEdge.
(TIF)

## Abbreviations

IMJ Edge: ImageJ macro Cell Colony Edge
IMJ Cai: ImageJ macro by Cai et al.
TIC: Tumor initiating cell
MCTS: Multicellular Tumor Spheroids
ROI: Region of Interest
