# Supplementary material for: High-Throughput Method for Automated Colony and Cell Counting by Digital Image Analysis Based on Edge Detection
Source: PLoS One. 2016 Feb 5;11(2):e0148469. doi: 10.1371/journal.pone.0148469 (PMC4746068; doi:10.1371/journal.pone.0148469)
Supplement: S1 Appendix — The macro given in text. The text in green explains the function of the following code. The parameters used for different images are also given. (PDF) [file pone.0148469.s001.pdf]

S1 Appendix

ImageJ Cell_Colony_Edge Macro

```
//This macro automatically detects, counts and measures cells and colonies in a folder of images.

//It requires the user to specify input folder (containing only images) and output folder (where

// results are stored.  In addition the macro writes new images displaying outlines of measured

//particles in the output folder. Parameters such as minimum and maximum particle size etc are

//determined manually by the user and entered at the prompts provided by the macro.

//Use the Analyze>Set Measurements command to specify the measurements

//that are recorded for each cell/colony.


macro Cell_Colony_Edge{


//Displays prompt for setting scale to values determined from calibration slide. Default value is 0
and pixel, which leave the scale in pixels as is.

Dialog.create("Type and Set Scale")

        Dialog.addNumber("Number of pixels/unit", 0);

        Dialog.addChoice("Unit:", newArray("pixel", "um"));

        Dialog.show();

        n = Dialog.getNumber();

        u = Dialog.getChoice();



//Displays prompt for entering predetermined parameter values

Dialog.create("Parameters")

        Dialog.addCheckbox("Subtract Background", true);

        Dialog.addNumber("Rolling Ball Radius:", 50);

        Dialog.addNumber("Remove Outliers 1-radius:", 0.25);

        Dialog.addChoice("Remove Outliers 1 color:", newArray("Bright", "Dark"));

        Dialog.addNumber("Gaussian Blur-sigma:", 2);

        Dialog.addNumber("Remove Outliers 2-radius:", 8);
```

```
		Dialog.addNumber("Pixel Maximum:", 2);
		Dialog.addNumber("Pixel Minimum:", 3);
		Dialog.addCheckbox("Watershed", true);
		Dialog.addNumber("Remove Outliers 3-radius:", 12);
		Dialog.addNumber("Analyze Particles - Min size:", 50);
		Dialog.addNumber("Analyze Particles - Min circ:", 0.2);
		Dialog.addCheckbox("Measure Intensity from original image", false);
		Dialog.show();
//Assigns the entered values to variables
sub = Dialog.getCheckbox();
br = Dialog.getNumber();
or1 = Dialog.getNumber();
col = Dialog.getChoice();
s = Dialog.getNumber();
or2 = Dialog.getNumber();
mxr = Dialog.getNumber();
mnr = Dialog.getNumber();
wtr = Dialog.getCheckbox();
or3 = Dialog.getNumber();
mnsz = Dialog.getNumber();
mncr = Dialog.getNumber();
orig = Dialog.getCheckbox();

//Displays Prompt for selection of Input & Output Directory
Idir = getDirectory("Choose Input Directory ");
Odir = getDirectory("Choose Output Directory");

list = getFileList(Idir);
if (getVersion>="1.40e")
	setOption("display labels", true);
```

```
setBatchMode(true);

for (i=0; i<list.length; i++) {

	showProgress(i, list.length);

	processFile(Idir, Odir, list[i]);

				}




//Saves results and summary in Output Directory

selectWindow("Results");

saveAs("Measurements", ""+Odir+"Results.txt");

selectWindow("Summary");

saveAs("Text", ""+Odir+"Summary.txt");



function processFile(Idir, Odir, filename)

		{

		open(Idir + filename);

		run("Set Scale...", "distance=n known=1 pixel=1 unit=u global");


		//Subtracts Backgound. To skip this step, deselect option in prompt.

		if (sub==true){

				run("Subtract Background...", "rolling=br light separate");

				}




		//Sharpens, enhances and finds edges in image

		run("Sharpen");

		run("Enhance Contrast...", "saturated=0.2");

		run("Remove Outliers...", "radius=or1 threshold=0 which=col");
```

```
run("Find Edges");



//Smoothens and makes the image Black and white

run("Gaussian Blur...", "sigma=s");

run("Make Binary");



//Closes and Fills Holes. Remove outliers step is for denoising and eliminating
//debris particles. Can set size 0 in prompt if step is unnecessary.

run("Close-");

run("Fill Holes");

run("Remove Outliers...", "radius=or2 threshold=0 which=Dark");



//Expand pixels to fill holes, and shrink back to normal size

run("Maximum...", "radius=mxr");

run("Close-");

run("Fill Holes");

run("Minimum...", "radius=mnr");



//Denoising to eliminate debris and unwanted particles. Size of outliers is set
//in prompt. Watershed command separates fused cells/colonies

run("Despeckle");

if (wtr==true){

        run("Watershed");

        }

run("Remove Outliers...", "radius=or3 threshold=0 which=Dark");
```

```
}

    //Analyze particles to measure highlighted objects. Minimum and Maximum sizes,

    //and circularities can be chosen in the prompt.

    roiManager("Show All with labels");

    roiManager("Show All");

    if (orig==true)

        run("Analyze Particles...", "size=mnsz-Infinity circularity=mncr-1.00 show=[Overlay
Outlines]  exclude summarize add");

     else

    run("Analyze Particles...", "size=mnsz-Infinity circularity=mncr-1.00 show=[Overlay
Outlines] display exclude summarize add");



    //Sends outlines from processed binary image to the original image via the ROI manager.
    Saves the original image displaying outlines in the output directory.

    run("From ROI Manager");

    close();

    open(Idir + filename);

    run("From ROI Manager");

    roiManager("Show All with labels");

    roiManager("Show All");

    if (orig==true) {

        roiManager("Measure");

    }

    Opath = Odir + filename;

    saveAs(“JPEG”, Opath);

    close();


    }


}
```

Parameters for Tumorsphere measurement

n = 0.48;

u = um;

sub = true;

br = 80;

or1 = 0;

s = 2;

or2 = 8;

mxr = 2;

mnr = 3;

or3 = 12;

mnsz = 700;

mncr = 0.2;

Parameters for Clonogenic assay measurement

n = 0;

u = pixel;

sub = true;

br = 50;

or1 = 0;

s = 0.5;

or2 = 8;

mxr = 2;

mnr = 3;

or3 = 12;

mnsz = 50;

mncr = 0.2;

Parameters for Bacterial Colony measurement

n = 0;

u = pixel;

sub = false;

br = n/a;

or1 = 2;

s = 1;

or2 = 8;

mxr = 1;

mnr = 4;

or3 = 5;

mnsz = 75;

mncr = 0.2;
