# Supplementary material for: High-Throughput Method for Automated Colony and Cell Counting by Digital Image Analysis Based on Edge Detection
Source: PLoS One. 2016 Feb 5;11(2):e0148469. doi: 10.1371/journal.pone.0148469 (PMC4746068; doi:10.1371/journal.pone.0148469)
Supplement: S2 Appendix — (PDF) [file pone.0148469.s002.pdf]

# S2 Appendix

## Customized and simplified versions of Cell_Colony_Edge Macro for specific purposes.

### IMJ Edge for Bacterial Colonies (Folder of Images)

```
dir = getDirectory("Choose Input Directory ");
list = getFileList(dir);
if (getVersion>="1.40e")
    setOption("display labels", true);
setBatchMode(true);
for (i=0; i<list.length; i++) {
    path = dir+list[i];
    showProgress(i, list.length);
    open(path);
    processFile(path);
    close();
        }


function processFile(path){

        run("Sharpen");
        run("Despeckle");
        run("Enhance Contrast...", "saturated=0.2");
        run("Remove Outliers...", "radius=2 threshold=0 which=Bright");
        run("Find Edges");
        run("Make Binary");
        run("Gaussian Blur...", "sigma=1");
        run("Make Binary");
        run("Remove Outliers...", "radius=2 threshold=0 which=Dark");
        run("Fill Holes");
        run("Despeckle");
        run("Watershed");
        run("Analyze Particles...", "size=2-Infinity circularity=0.20-1.00 show=[Overlay Outlines]
        display exclude add");
        roiManager("Show All with labels");
        roiManager("Show All");
        }
```

### IMJ Edge for Clonogenic Assay (Single Image)

```
        run("Subtract Background...", "rolling=50 light");
        run("Sharpen");
        run("Enhance Contrast...", "saturated=0.2");
        run("Find Edges");
        run("Make Binary");
        run("Despeckle");
```

```
run("Gaussian Blur...", "sigma=0.5");
run("Make Binary");
run("Close-");
run("Fill Holes");
run("Remove Outliers...", "radius=8 threshold=0 which=Dark");
run("Maximum...", "radius=1");
run("Close-");
run("Fill Holes");
run("Minimum...", "radius=4");
run("Despeckle");
run("Watershed");
run("Remove Outliers...", "radius=5 threshold=0 which=Dark");
run("Analyze Particles...", "size=75-Infinity circularity=0.20-1.00 exclude add");
roiManager("Show All with labels");
roiManager("Show All");
run("From ROI Manager");
close();
open("ORIGINAL FILE");  //Give full path here
run("From ROI Manager");
roiManager("Show All with labels");
roiManager("Show All");
roiManager("Measure");
```
