# Supplementary material for: High-Throughput Method for Automated Colony and Cell Counting by Digital Image Analysis Based on Edge Detection
Source: PLoS One. 2016 Feb 5;11(2):e0148469. doi: 10.1371/journal.pone.0148469 (PMC4746068; doi:10.1371/journal.pone.0148469)
Supplement: S3 Appendix — (PDF) [file pone.0148469.s003.pdf]

S3 Appendix

Manual determination of parameters using ImageJ GUI_(Note the different Remove Outlier steps, and their placement within the macro)

1. Open image
2. Image → Duplicate
    a. Enter title
3. Process → Subtract Background
    a. Select option 'Preview' to test out different radii, conditions etc
    b. Enter value for radius of rolling ball (near average diameter of objects)
    c. Select option 'light background'
    d. Select option 'Separate' if RGB image
4. Process → Sharpen
5. Process → Noise → Remove Outliers 1 (if needed)
    a. Select option 'Preview' to test out different radii
    b. Enter value '0' for Threshold
    c. Select option "Bright' or 'Dark' for Which Outliers (depending of color of debris)
    d. Enter value for radius (to exclude debris and background, while keeping cells and colonies). Enter value 0 if not needed.
6. Process → Enhance Contrast
    a. Enter value '0.2' for Saturated Pixels
7. Process → Find Edges
8. Process → Binary → Make Binary
9. Process → Filters → Gaussian Blur
    a. Select option 'Preview' to test out different radii
    b. Enter value for radius (to smooth out colony edges)
10. Process → Binary → Make Binary
11. Process → Binary → Close
12. Process → Binary → Fill Holes
13. Process → Noise → Remove Outliers 2
    a. Select option 'Preview' to test out different radii
    b. Enter value '0' for Threshold
    c. Select option "Dark' for Which Outliers
    d. Enter value for radius. Compare to the original image to ensure real colonies are not excluded. Enter value 0 if not needed.
14. Process → Filters → Maximum
    a. Select option 'Preview' to test out different radii
    b. Enter value for radius (to close circles for cells and colonies)
15. Process → Binary → Close
16. Process → Binary → Fill Holes
17. Process → Filters → Minimum
    a. Select option 'Preview' to test out different radii
    b. Enter value for radius (to return object sized to original)

18. Process → Noise → Remove Outliers (if needed)
19. Process → Binary → Watershed (if needed. Skip if measuring tube assay)
20. Process → Noise → Remove Outliers 3
21. Analyze → Set Measurements
    a. Select measurements such as Area
    b. Select options 'Add to overlay', and 'Display label'
    c. Select the original image name from the 'Redirect to' pulldown menu
22. Analyze → Analyze Particles
    a. Enter range of values for Size and Circularity
    b. Select 'Outlines' in Show pulldown menu
    c. Select options 'Display results', 'Summarize', 'Add to Manager', 'Exclude on edges'
23. Select the Result window. File → Save as → select folder, and enter filename.
24. Similarly save processed image (if needed)
