# Supplementary material for: High-Throughput Method for Automated Colony and Cell Counting by Digital Image Analysis Based on Edge Detection
Source: PLoS One. 2016 Feb 5;11(2):e0148469. doi: 10.1371/journal.pone.0148469 (PMC4746068; doi:10.1371/journal.pone.0148469)
Supplement: S4 Appendix — (PDF) [file pone.0148469.s004.pdf]

S4 Appendix

Instructions for downloading and running the macro

1. Download and install ImageJ:
   http://rsbweb.ih.gov/ij/download.html
2. Download the macro Cell Colony Edge. Or copy-paste from Appendix A, and save as a .txt file
3. Place the Cell_Colony_Edge.txt file into the directory containing the folder of images
4. To launch the macro: Plugins → Macros → Run
   Navigate to and select the macro file, and click 'Open'
5. Enter values for scale in popup
   a. Select Unit as pixel or um
   b. Enter value for 'Number of pixels/unit' if scale is known.
   c. If scale is not known, leave as 0 and unit as pixel. Image will be left unchanged
   d. Press OK
6. Enter parameter values in popup
   a. Select option 'Subtract Background' if needed
   b. Enter parameter values (manually determined as per steps in Appendix C).
7. Check the box for original image, if Intensity measurements are to be made from original image rather than the processed image
8. Select Input Directory (containing only images) and press 'Choose'
9. Select location of Output Directory using the popup, and press 'Choose'
10. Let macro run, process all the files, and save outlined copies in the Output Directory
11. Open files in the Output Directory with ImageJ to confirm correct determination of cells and colonies
12. Analyze Results.txt as needed.
