# Supplementary figures and images for: High-Throughput Method for Automated Colony and Cell Counting by Digital Image Analysis Based on Edge Detection
Source: PLoS One. 2016 Feb 5;11(2):e0148469. doi: 10.1371/journal.pone.0148469 (PMC4746068; doi:10.1371/journal.pone.0148469)

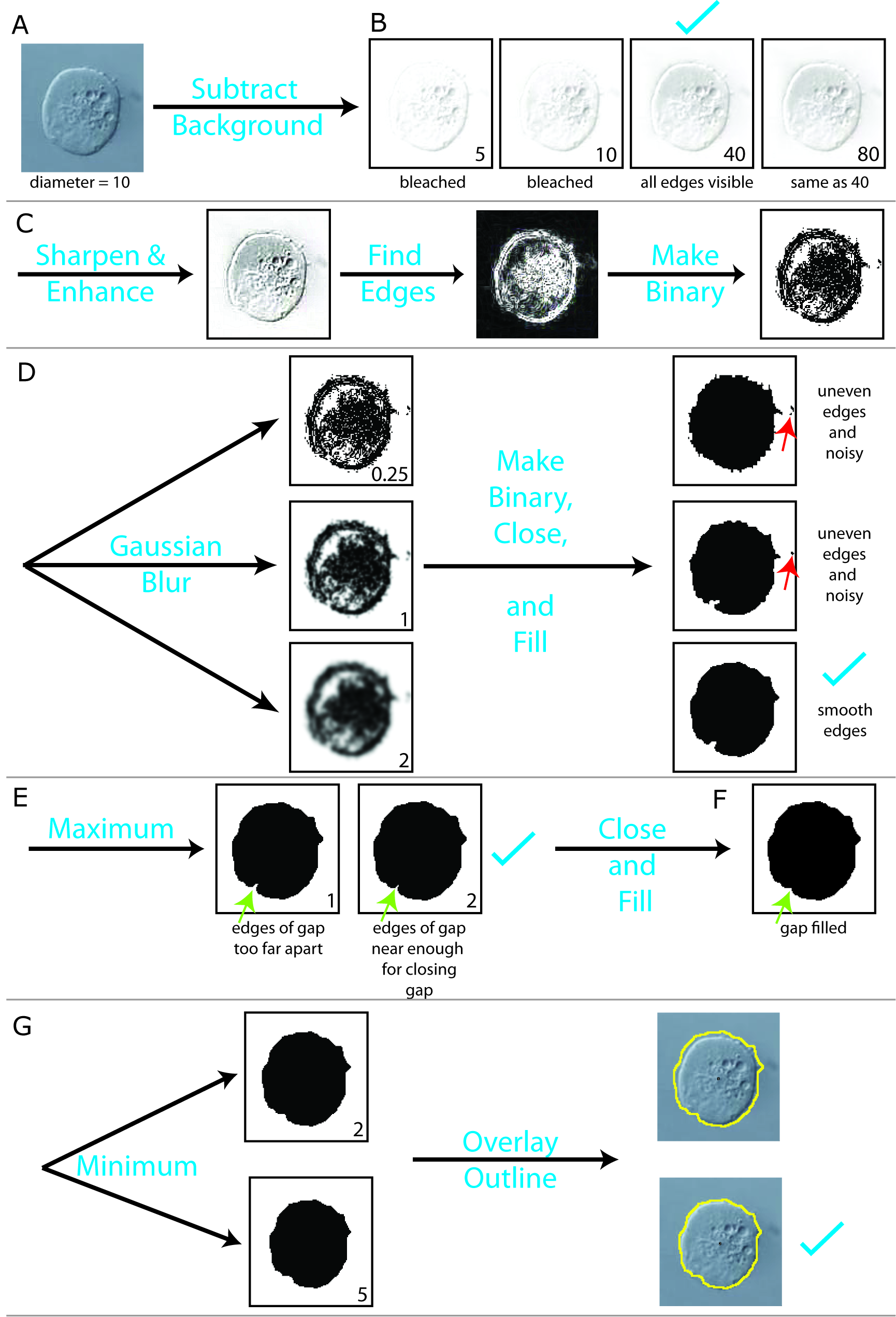

Supplement: S1 Fig — (Figure A) Original Nomarksi image showing a single U266 cell (modified from [40]). The diameter was measured as 10 px in ImageJ. (Figure B) Image in (A) after background subtraction with different rolling ball radii (written in bottom right corner of each image). Rolling ball radius of 40 was selected (blue checkmark) because all edges were visible. (Figure C) Background subtracted image is then sharpened and enhanced (0.2%). This image is further processed by the “Find Edges” and “Make Binary” commands. (Figure D) The binary image is processed by the “Gaussian blur” command with different sigma radii (written in bottom right corner of each image), followed by filling and closing of holes. Red arrows show that debris is processed as an object when 0.25 or 1 is used as radius for Gaussian blur. Gaussian blur with radius 2 did not select debris as an object, and was selected (blue checkmark). (Figure E) Closed and Filled image in D is further processed by the “Maximum” command to increase size of each pixel. This brings the edges of gaps closer together (green arrows). Radius 2 was selected, as the edges of gap are close enough for filling and closing. (Figure F) Image in E was closed and filled. (Figure G) Size of pixels is reduced back. Radius 5 is chosen because size of selection is similar to the size of cell in original image. Overlay is shown for comparison purposes. (TIFF) [file pone.0148469.s005.tiff]

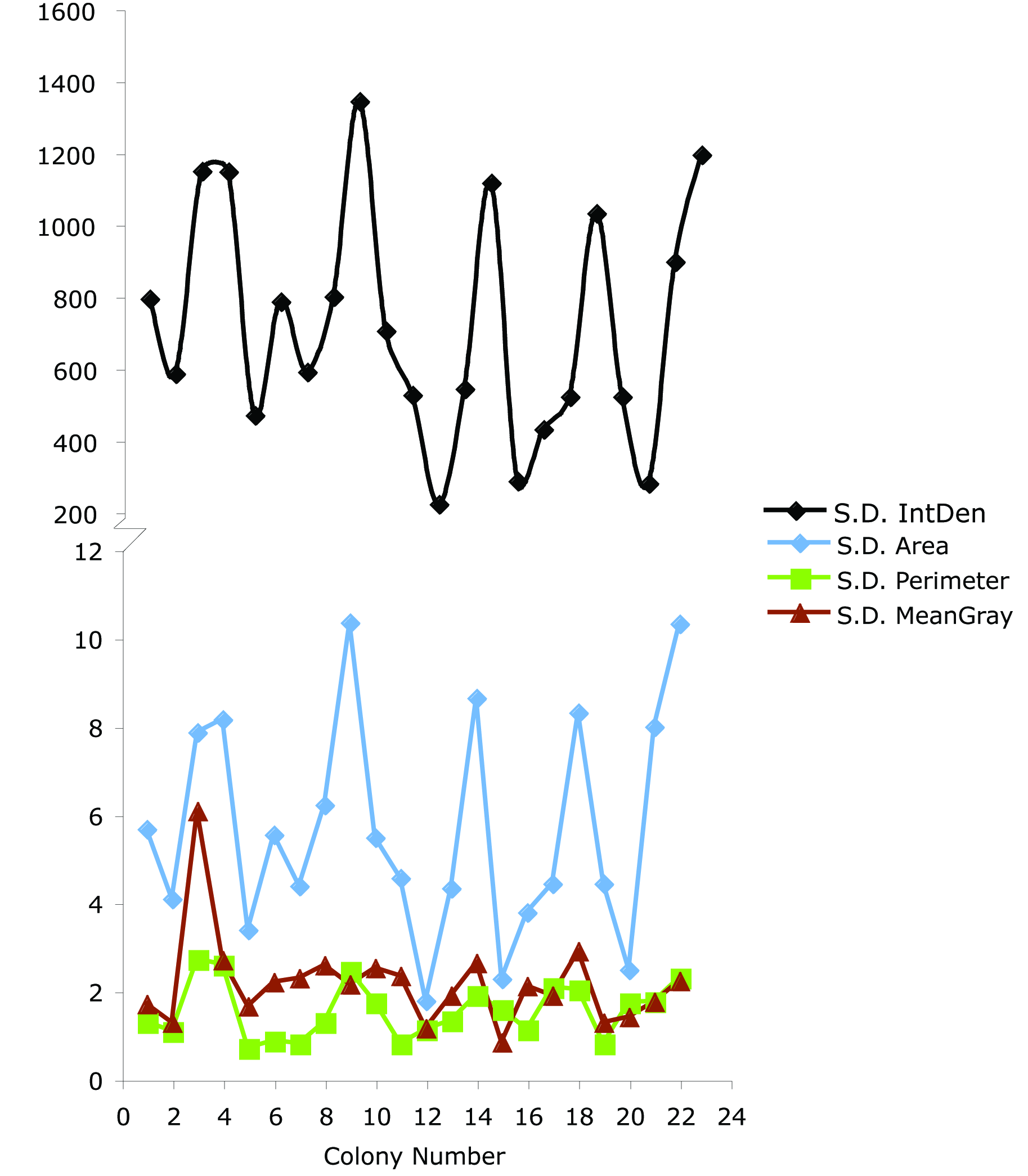

Supplement: S2 Fig — The plot is broken in two halves with different scales to represent different values on the y-axes with Colony numbers on the x-axis. (TIF) [file pone.0148469.s006.tif]

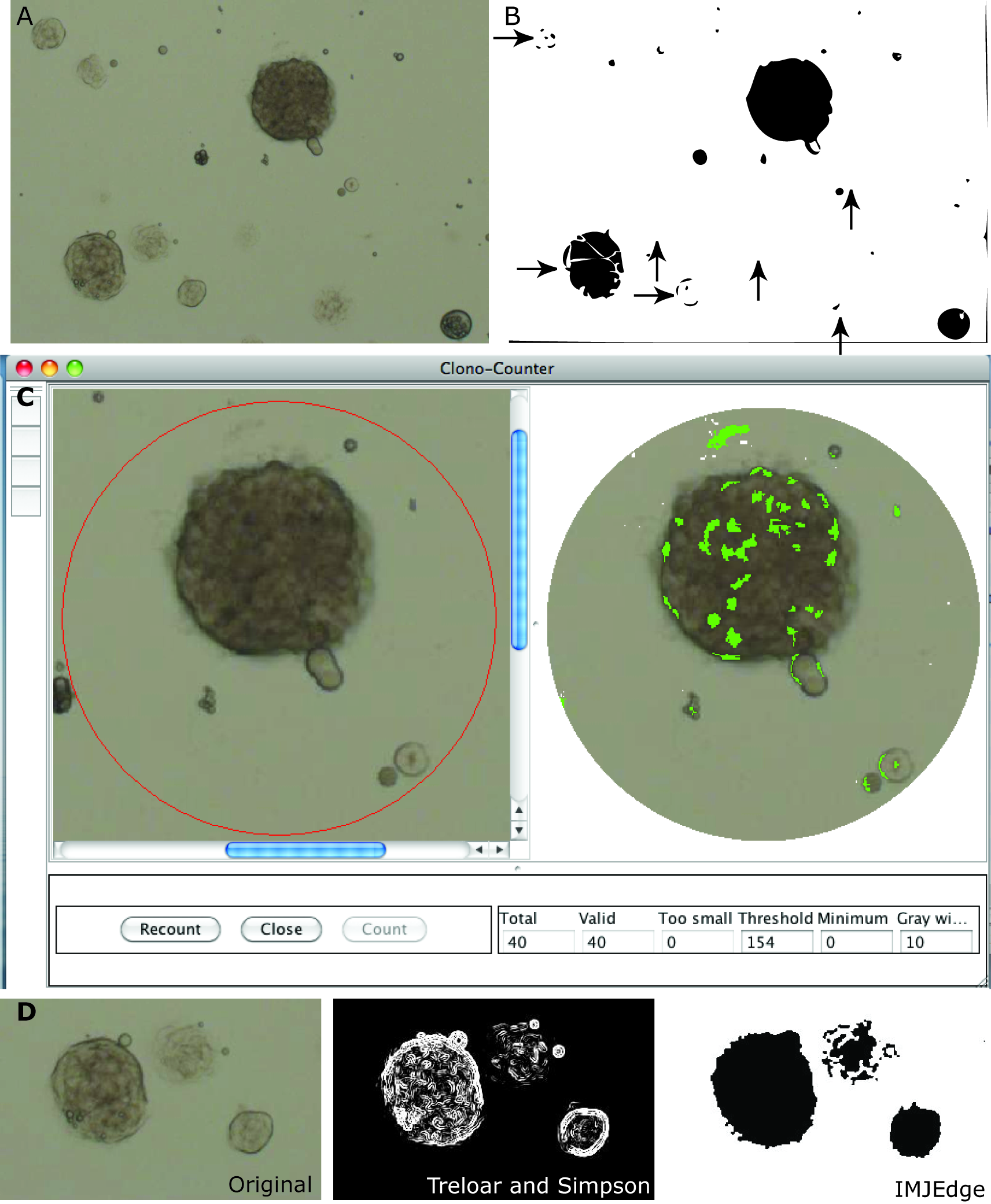

Supplement: S3 Fig — (Figure A) Original image of tumorspheres processed by different methods. (Figure B) Results of image processed by Sieuwerts et al ImageJ plugin. Vertical arrows point to undetected colonies, and horizontal to colonies that are only partly detected. (Figure C) Image of Clonocounter working on Image in Figure A. Only part of the image inside the circle is analyzed. In this part too, the green highlights show the detected colonies. The table underneath shows the results of clonocounter. (Figure D) Original tumorsphere image on the left followed by the image processed by Treloar and Simpson ImageJ method and by IMJEdge. (TIF) [file pone.0148469.s007.tif]
